# The rocky relationship between global warming and ocean biodiversity: an interview with Rui Seabra

**DOI:** 10.1038/s42003-023-05597-1

**Published:** 2023-12-17

## Abstract

Dr. Rui Seabra studies the thermal landscapes of rocky shores across the world and how environmental complexity drives species’ distributions and vulnerability to global warming. He co-leads the Coupled Coastal Temperature and Biodiversity Observation Network (CCTBON), a network aimed at monitoring patterns of rocky shore temperatures and biodiversity patterns across the Atlantic Ocean. In this Q&A we discuss the challenges of biodiversity research at the global scale.

**Figure:**
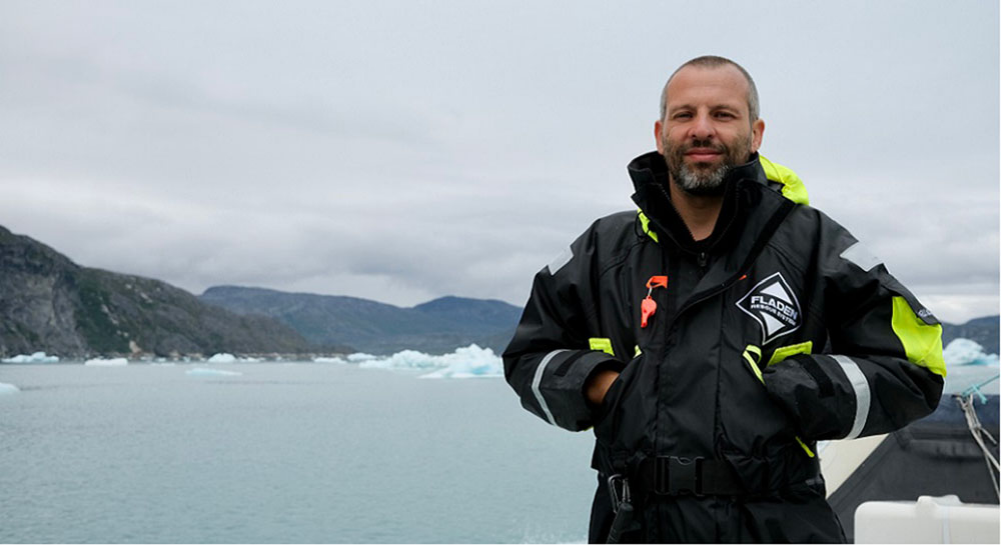
Ana Catarina Queiroga. Pictured: Rui Seabra

Can you tell us about your current research?

Ever since my Ph.D I’ve been studying the thermal landscape of rocky shores and how it shapes biodiversity patterns. Rocky shores are a particularly interesting model ecosystem for the study of how biodiversity responds to environmental extremes and a changing climate because tides and waves make them very environmentally complex and because intertidal species often live close to their physiological limits.

When we think of monitoring ocean temperature at the global scale, remote sensing technology like satellites immediately comes to mind. But, while remotely sensed data will always be crucial for large-scale studies or where no other data are available, over the last decade intertidal ecologists have become increasingly aware that many important patterns only arise at small scales of under a meter. For example: lizards can hide from the heat if shade is available so you can’t rely on data averaged out over too vast areas. The same is true on rocky shores, where the continuous cycling of tides means conditions are constantly transitioning between terrestrial and marine — a degree of complexity that just simply cannot be captured using remotely sensed data.

For this reason — and as much as many people have tried otherwise — we must monitor intertidal environments and biodiversity locally. Currently, together with my colleague Fernando Lima, and a team of fantastic biology post-docs, hardware, and software engineers, I’m leading the implementation of the largest-ever temperature and biodiversity monitoring network focused on rocky intertidal ecosystems. Over the next 3–4 years, with the Coupled Coastal Temperature and Biodiversity Observation Network (CCTBON) we’ll be installing 2000 temperature sensors in the mid-intertidal portion of rocky shores and in more than 200 sites spread across the entire Atlantic Ocean, from Pole to Pole and encompassing the East and West continental coastlines as well as the majority of the oceanic islands (additional info: www.coastalwarming.com/atlantic-cctbon) and surveying biodiversity regularly, in collaboration with local researchers.

The current focus is on the Atlantic Ocean because it’s the smallest of the only two oceans that encompass the full range of latitudes, climates, and tidal regimes (the other is the vastly larger Pacific Ocean). However, as we refine our tools and methodologies, wider networks will become increasingly feasible, at which point we will undoubtedly aim towards a truly global intertidal monitoring effort.

Why is looking at temperature changes in the coastal and/or intertidal areas so important for biodiversity and how does it affect oceanic sustainability?

While coastal areas only represent a very small fraction of the area covered by the world’s oceans, they are home to a disproportionate amount of their biodiversity and productivity, certainly much more than the open ocean. Coastal regions are also important nurseries and feeding grounds for many marine species (including species with economic value) and play a defining role in many societies’ cultures. Rocky shores, specifically, are home to complex communities of organisms that, on top of all else, are especially vulnerable to Climate Change impacts, making them the proverbial “canary in the coal mine” that societies should be keeping a watch over.

There is increasing awareness that, when selecting areas for conservation, one must consider not only how biodiverse a given site is but also how likely it is that the underlying environmental conditions will remain sufficiently stable into the future — or else we may end up trying to conserve a place that is doomed by impending climatic changes. Pinpointing the right locations in which to invest a greater societal effort toward conservation, as well as those where mitigation efforts should focus more on rearranging local economies to better match future unavoidable changes to biodiversity patterns, is fundamental to the sustainable use of the oceans.

But identifying those areas is easier said than done, especially along the world’s coastlines. For a long time, researchers have relied on remotely sensed temperatures for large-scale studies but work from my team has shown that on many coastlines, estimates of sea surface temperature often miss the mark by a lot when compared to in situ measurements (up to 6 °C too hot in shores in areas with strong upwelling of cold bottom water). Furthermore, the ebb and flow of the tides expose intertidal organisms to a cycle of terrestrial and marine conditions. Thermal inertia and many other environmental factors mean that the resulting thermal profile cannot be accurately reconstructed from separate measurements of air and sea surface temperature, especially if derived from remotely sensed products. This is why I’m a strong advocate for the in situ monitoring of environmental conditions on rocky shores (or any other ecosystem where strong thermal landscape heterogeneity arises at scales of under a meter) and why all my monitoring efforts nowadays rely on that approach. And then there’s the question about which features of the temperature experienced by an organism over time actually matter for performance and survival (mean, extremes, etc.), from the individual to the species level. The better we get at understanding these factors, the more effective conservation efforts will be — on rocky shores and elsewhere.

Your research involves collaborations with researchers from around the globe, from Guinea-Bissau to Norway. Can you tell us about the challenges and rewards of having a diverse group of researchers?

It is a fact that this Atlantic network relies heavily on local researchers, much more than any other network I have implemented before. The reason for that is twofold. First, there’s the unavoidable need for practicality and cost efficiency. One single team just simply cannot visit shores across an entire ocean on a regular basis, which is what is required for the collection of the target biodiversity dataset. You can pull a one-off visit to many of the sites—as I am attempting to do—but to go back again and again is totally impractical. However, the local researchers we engage with are typically already involved in long-term monitoring efforts in their region and, therefore, can return to the sites more easily and frequently.

Second, we want this network to be a tool for the empowerment of local researchers, especially in chronically underfunded countries. This means that local researchers must be part of several steps of the process, from site selection to data collection and analysis. By installing temperature sensors and providing a rugged smartphone and additional equipment to our collaborating researchers, the CCTBON deploys infrastructure without cost to local researchers that, together with the data it generates, will facilitate their participation in wider-scoped studies, likely leading to more impactful research, and ultimately boosting their careers. The smartphone apps that we’ve been developing in partnership with ElectricBlue (a startup co-op I co-founded; www.electricblue.eu) have been game-changers here because they streamline the tracking of all data a user contributes (much like the GenBank model), ensuring that all collaborating researchers get the recognition they deserve.

Of course, there are challenges in relying on so many actors. Cultural differences generate barriers that require effort and social awareness to overcome — typical sources of friction revolve around the flexibility of schedules (sometimes too little, other times too much), level of commitment, methodological disagreements, and others. But I’m yet to find an expedition where such issues remain unresolved, and so far, the experience has been overwhelmingly positive. In most expeditions, I’ve easily managed to establish strong links with the researchers and/or park rangers (and other stakeholders) who host/accompany me, ensuring they become the proud custodians of the new sites we install together. It’s also very common for new student/research collaborations to be forged during commuting or post-fieldwork meals. As a consequence, my network of professional contacts has expanded many-fold over the past years, which is invaluable for my career as well. Even though I hadn’t reflected on the human side of this entire experience beforehand, I’m constantly amazed at the number of inspiring people I keep meeting because of the CCTBON, and I’m very grateful for that.

The transition from the two-person network we were running during my Ph.D. to the truly collaborative CCTBON has been really eye-opening to me, and I’m now looking at the next step in this push to democratize science and the scientific experience. One of the main distinctive features of this network is that the methods employed are simple and fully standardized. The simplicity of many of these tasks opens the door to enlisting citizens for data collection, and that’s something we are actively working on now.

How do you see your research shaping future decisions in ocean sustainability?

Realistically speaking, the most relevant outputs of this effort will only come within a decade. Once that time has lapsed, we’ll have compiled the two full-ocean catalogs of intertidal biodiversity, and we’ll have collected 10 years of hourly temperature data at the microhabitat level from hundreds of sites.

Considering that presently there are vast lengths of Atlantic coastline virtually absent from published research on intertidal ecology, especially from the “forgotten” tropical Atlantic, the surveys of biodiversity will alone represent a major contribution. With these catalogs and the temperature data, we will be in a commanding position to redefine the distribution limits of many species, map temperature and biodiversity change with unprecedented detail, and study the link between the two at a truly oceanic scale. These, in turn, will allow the identification of biodiversity hotspots and climate change refugia across the entire Atlantic, providing crucial information for communities, stakeholders, and policymakers engaged in biodiversity management, conservation, and societal adaptation. The large-scale nature of these outputs will also facilitate a broader contextualization of regional patterns and hopefully lead to transnational cooperation. Another measure of success will be the adoption of this framework for the monitoring of other ecosystems, such as rivers, forests, or the deep sea—I already have active collaborations in that direction. Ultimately, my hope is that our contributions eventually get picked up by international institutions such as the UN and its IPCC panel, providing a unique insight into the health of coastal regions over a vast area.

Besides research, you are involved in many other projects related to the sea. What is the origin of your passion for the Ocean?

I’ve always lived in Praia de Angeiras, a fishing village north of Porto, in northern Portugal. My parental grandmother was one of the many brave women who endured cold and danger to harvest laminaria for the cosmetic industry since she was a child. My grandmother’s father was in charge of the local lifeboats at a time when rescues were about as dangerous as they could be. My father is a key figure in lifesaving and drowning prevention nationally and internationally. I began swimming at a very young age, and I’m an avid wave rider. Curiously, my wife, who had none of this family sea tradition, eventually became a leading researcher in the field of drowning prevention and is currently working with the WHO on that topic. Inevitably, over the years, I have also become versed in drowning prevention, and I’m a coauthor on papers on the matter. More recently, my wife has also become involved with the Surfrider Foundation, an international NGO promoting safe and clean coastal environments. I have joined along, and together with other NGOs, we’ve been engaging communities and politicians to improve the health of our beaches. So yes, you could say that I like to be by the sea.

*This interview was conducted by Associate Editor Joao Valente*.

